# An isolated water droplet in the aqueous solution of a supramolecular tetrahedral cage

**DOI:** 10.1073/pnas.2012545117

**Published:** 2020-12-14

**Authors:** Federico Sebastiani, Trandon A. Bender, Simone Pezzotti, Wan-Lu Li, Gerhard Schwaab, Robert G. Bergman, Kenneth N. Raymond, F. Dean Toste, Teresa Head-Gordon, Martina Havenith

**Affiliations:** ^a^Lehrstuhl für Physikalische Chemie II, Ruhr-Universität Bochum, 44780 Bochum, Germany;; ^b^Chemical Sciences Division, Lawrence Berkeley National Laboratory, University of California, Berkeley, CA 94720-1460;; ^c^Department of Chemistry, University of California, Berkeley, CA 94720-1460;; ^d^Pitzer Center for Theoretical Chemistry, University of California, Berkeley, CA 94720-1460

**Keywords:** supramolecular, encapsulation, THz spectroscopy, ab initio molecular dynamics, confined water

## Abstract

Water under nanoconfinement at ambient conditions has exhibited low-dimensional ice formation and liquid–solid phase transitions, but with structural and dynamical signatures that map onto known regions of water’s phase diagram. Using terahertz (THz) absorption spectroscopy and ab initio molecular dynamics, we have investigated the ambient water confined in a supramolecular tetrahedral assembly, and determined that a dynamically distinct network of 9 ± 1 water molecules is present within the nanocavity of the host. The low-frequency absorption spectrum and theoretical analysis of the water in the Ga_4_L_6_^12−^ host demonstrate that the structure and dynamics of the encapsulated droplet is distinct from any known phase of water. A further inference is that the release of the highly unusual encapsulated water droplet creates a strong thermodynamic driver for the high-affinity binding of guests in aqueous solution for the Ga_4_L_6_^12−^ supramolecular construct.

## Significance

Using a supramolecular assembly that catalyzes substrates in water, we show that the nanocage encapsulates a water cluster that is structurally and dynamically distinct from any known phase of water. It plays an important role in the driving force for guest encapsulation: The release of the highly unusual encapsulated water droplet creates a strong thermodynamic drive for the high-affinity binding of guests in aqueous solution for the supramolecular construct.

Supramolecular capsules create internal cavities that are thought to act like enzyme active sites (1). As aqueous enzymes provide inspiration for the design of supramolecular catalysts, one of the goals of supramolecular chemistry is the creation of synthetic “receptors” that have both a high affinity and a high selectivity for the binding of guests in water (2, 3). The Ga_4_L_6_^12−^ tetrahedral assembly formulated by Raymond and coworkers represents an excellent example of a water-soluble supramolecular cage that has provided host interactions that promotes guest encapsulation. Using steric interactions and electrostatic charge to chemically position the substrate while shielding the reaction from solvent, this host has been shown to provide enhanced reaction rates that approach the performance of natural biocatalysts (4–10). Moreover, aqueous solvation of the substrate, host, and encapsulated solvent also play an important role in the whole catalytic cycle. In particular, the driving forces that release water from the nanocage host to favor the direct binding with the substrate is thought to be a critical factor in successful catalysis, but is challenging to probe directly (7, 8, 11–14).

In both natural and artificial nanometer-sized environments, confined water displays uniquely modified structure and dynamics with respect to the bulk liquid (15–18). Recently, these modified properties were also found to have significant implications for the mechanism and energetics of reactions taking place in confined water with respect to those observed in bulk aqueous solution (19–21). In a pioneering study on supramolecular assemblies, Cram and collaborators (22) concluded that the interior of those cages is a “new and unique phase of matter” for the incarcerated guests. In more recent studies, it was postulated that, similar to graphitic and zeolite nanopores (23, 24), confined water within supramolecular host cavities is organized in stable small clusters [(H_2_O)_*n*_, with *n* = 8 to 19] that are different from gas phase water clusters (25). In these studies, the hydrogen-bonded water clusters were reported to be mostly ice- or clathrate-like by X-ray and neutron diffraction in the solid state at both ambient and cryogenic temperatures (26–32). However, to the best of our knowledge, such investigations have not characterized the Ga_4_L_6_^12−^ supramolecular tetrahedral assembly in the liquid state near room temperature and pressure, where the [Ga_4_L_6_]^12−^ capsule can perform catalytic reactions (6, 8, 9).

Here, we use terahertz (THz) absorption spectroscopy and ab initio molecular dynamics (AIMD) to characterize low-frequency vibrations and structural organization of water in the nanoconfined environment. THz is ideally suited to probe the intermolecular collective dynamics of the water hydrogen bond (HB) network with extremely high sensitivity, as illustrated for different phases of water (33–38), and for aqueous solutions of salts, osmolytes, alcohols, and amino acids (36, 39–42). The THz spectra of the water inside the nanocage has been quantitatively reproduced with AIMD, allowing us to confidently characterize the water network in the cage in order to provide a more complete dynamical, structural, and thermodynamic picture. We have determined that the spectroscopic signature of the confined water in the nanocage is a dynamically arrested state whose structure bears none of the features of water at any alternate thermodynamic state point such as pressurized liquid or ice. Our experimental and theoretical study provides insight into the role played by encapsulated water in supramolecular catalysis, creating a low entropy and low enthalpy water droplet readily displaced by a catalytic substrate.

## Results

**Fig. 1.**
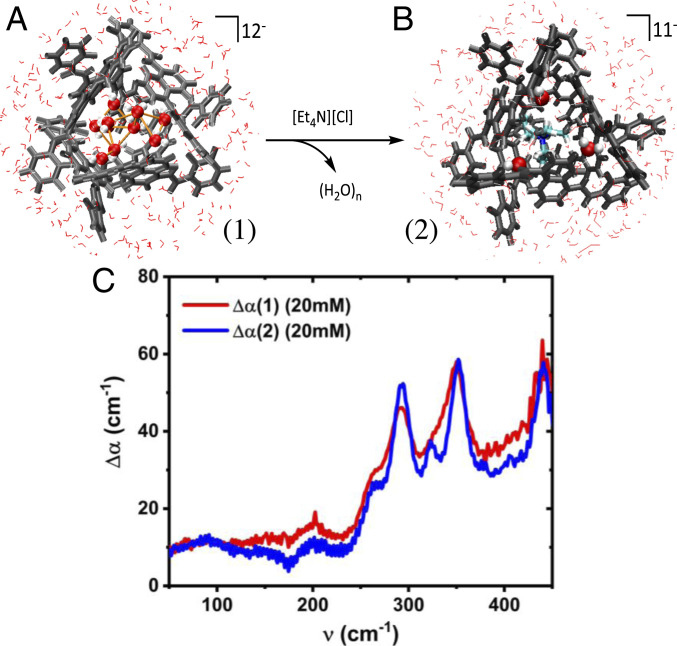
THz spectroscopy performed on the Ga_4_L_6_^12−^ tetrahedron with water vs. [Et_4_N]^+^ salt guests in the internal cavity. (*A*) Water molecules (oxygens in red, hydrogens in white) and the (*B*) cationic guest [Et_4_N]^+^ within the Ga_4_L_6_^12−^ tetrahedron. The HBs formed in-between water molecules inside the cage (orange lines) are also shown, as well as the three water molecules remaining within the cage in presence of the guest. In the Ga_4_L_6_^12−^ assembly, the metal ions occupy the four vertices and the ligands (L) are bridging aromatic spacers, occupying each of the six edges of the tetrahedron (gray bonds), and have a length of 12.9 Å. (*C*) Δα(ν) for water-filled (red line) and 20 mM [Et_4_N]^+^ guest-filled (blue line) inside the nanocage after bulk water subtraction. All of the absorption spectra were recorded under identical conditions (temperature, air humidity, and concentration). Details of the THz setup and difference spectra are provided in *SI Appendix*.

In the presence of strongly binding cationic salts such as tetraethylammonium salts ([Et_4_N]^+^) at a 1:1 guest:host concentration, all of the cations are quantitatively encapsulated in the cavity (*Materials and Methods*) given that their internal binding constant is almost three orders of magnitude larger than external binding, with minimal encapsulated water molecules present (Fig. 1*B*) (5, 6, 11, 14). At ambient conditions, the aqueous soluble naphthalene-based supramolecular host is proposed to contain some number of water molecules within the intramolecular space as well as interfacial solvent molecules associated with the external structure (Fig. 1*A*). However, the number and the nature of the water molecules encapsulated within the cage is not known, with or without the [Et_4_N]^+^ guest.

### Experimental Results.

The THz absorption spectra from ν = 50 to 450 cm^−1^ at 293 K were recorded for the water-filled and for the encapsulated [Et_4_N]^+^ guest at 20 mM (Fig. 1*C*), and a second concentration of 10 mM is reported in *SI Appendix*. These spectra were differenced from the bulk water spectrum to remove the contribution of the background solvent, and to determine the change in absorbance as a function of frequency, ∆*α*(*ν*) of the guest-filled complexes (for details, see *SI Appendix*). We find that the water-filled cavity displays an increased THz absorption with respect to that of the encapsulated salt in the 100 to 270 cm^−1^ range, which is characteristic of the changes in the intermolecular HB stretching of the water in and around the cage that differs from bulk water at ambient temperatures. In addition, peaks above 270 cm^−1^ were observed and assigned to the intramolecular modes of the Ga_4_L_6_^12−^ tetrahedral host that overlap with the broad librational band of water. Even so, we are only interested in the confined water signatures that occur at frequencies below 270 cm^−1^.

**Fig. 2.**
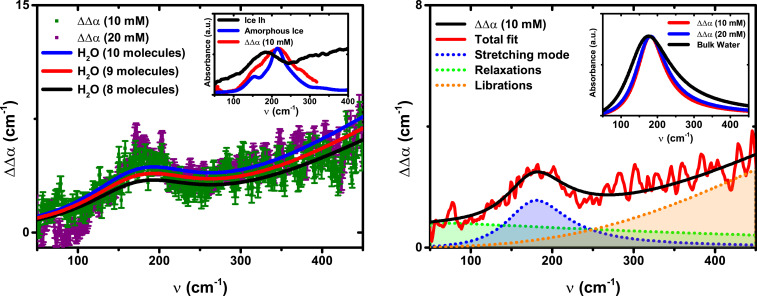
Double difference spectrum, ΔΔα(ν),of the THz fingerprint of the water cluster in the cavity of Ga_4_L_6_^12−^. (*A*) Experimental data at 10 mM (green points) and 20 mM (purple points) solutions, at 293 K. The data at 10 mM have been rescaled to the 20 mM concentration for comparison. (*Inset*) Absorbance spectra of ice Ih (blue line) from ref. 38, low-density amorphous ice (red line) from ref. 35, and ΔΔα(ν) at 10 mM. The maximum intensity of each spectrum has been normalized to unity for the sake of comparison. The error of the absorption coefficients of bulk water, hexagonal ice, and amorphous ice is less than 5%. (*B*) The different modes of the hydrogen-bonding network are dissected with a fit for intermolecular relaxation, HB stretching, and water librational modes (see text and *SI Appendix* for details). (*Inset*) Fit of the intermolecular stretching band of ΔΔα at 10 and 20 mM, respectively, and of bulk water at room temperature. All of the intensities have been rescaled to the maximum absorption of ΔΔ*α* at 10 mM.

Fig. 2 shows a double difference, ΔΔ*α*(*ν*), between the absorption of the guest–host complex in the presence [Δ*α*(2)(*ν*)] vs. absence [Δ*α*(1)(*ν*)] of the [Et_4_N]^+^ guest molecule to isolate the THz fingerprint of the water cluster in the cavity:

ΔΔα(ν)=Δα(1)(ν)−Δα(2)(ν). [1]

When the signal is normalized with respect to the host concentration, it is found that the intensity of ΔΔ*α*(*ν*) is independent of the host concentration of 10 or 20 mM. This provides validation that in these experiments that the number of water molecules inside the nanocage does not depend on the concentration of the supramolecular host, and indicates there is no aggregation or precipitation at the higher concentration.

The ΔΔ*α*(*ν*) intensity is then compared to a spectrum of bulk water that has been scaled by a number density to isolate the water count inside the cage. Inspection of Fig. 2*A* indicates that when compared against the bulk spectrum scaled by 8, 9, or 10 water molecules (black, red, and blue lines, respectively), it is proposed that 9 ± 1 H_2_O molecules are dynamically confined inside the cage in the absence of the salt (also see *SI Appendix*, Fig. S2). This is in agreement with the estimate for the number of water molecules that can be hosted in the cavity, taking into account a total volume of 270 Å^3^ (5). Even so, the estimated number of waters has to be considered as an average, resulting from the exchange of water molecules near the host interface with the bulk solvent through the open faces of the cage.

Not surprisingly, the spectrum of the encapsulated water shown in Fig. 2*A* is very different from the sharp absorption features observed for gas phase water clusters (43). Although the THz absorption spectroscopy is not a direct probe of the structure of a system, these low-frequency spectral signatures are specific fingerprints of the HB network of the isolated water cluster in the [Ga_4_L_6_]^12−^ host compared to other water systems (34, 38). In previous structural studies on supramolecular hosts in the solid state, encapsulated hydrogen-bonded (H_2_O)_8–10_ water clusters were identified to be similar to the smallest subunit of cubic ice (Ic) (26–28). Even after soaking a supramolecular host crystal in water for few hours, a crystallized water decamer was observed in the cavity, albeit without a perfectly close-packed arrangement as in ice (44). However, all these previous studies were of crystals and are not directly comparable to those carried out in solution under ambient conditions where the [Ga_4_L_6_]^12−^ capsule can perform catalytic reactions (6, 8, 9).

Thus, to better determine the nature of the encapsulated water, its spectrum was compared to those of hexagonal or amorphous ice (Fig. 2 *A*, *Inset*). The spectroscopic fingerprint of the confined water network lacks the characteristic peak at about 220 cm^−1^ with a shoulder at 150 cm^−1^ as observed in the case of Ic and hexagonal ice (Ih) (33, 38). In addition, the maximum of the band of water trapped inside the capsule is strongly redshifted with respect to the broad mode of low-density amorphous ice (at about 215 cm^−1^), indicating a weaker HB than the solid (35). Finally, the librational band of liquid water, i.e., the increased intensity above 250 cm^−1^, is clearly visible in ΔΔ*α*(*ν*), while it is missing in ice at these frequencies. Thus the spectrum of the encapsulated water does not resemble the spectrum of amorphous ice nor that of Ih or Ic.

To compare the similarity of capsule-confined water to other water phases, we performed a detailed analysis of the center frequencies, ${\tilde{\nu }}_{0}$, which indicate the strength of the HBs involved in the vibration, and linewidths, $w_{0}$, that yield information on the HB network with regards to lifetime and the number of available chemical environments (i.e., the degrees of freedom) (45). Therefore, the dynamics of the encapsulated water network, embodied in ΔΔ*α*(*ν*), was fitted to a sum of three damped harmonic oscillators, describing the relaxational, the intermolecular HB stretching, and the librational modes with increasing frequency (36). The resulting decomposed spectrum is shown in Fig. 2*B*.

The broad background extending to low frequencies (<100 cm^−1^) is attributed to dielectric relaxations and is found to be very similar to bulk water. The maximum of the librational peak (i.e., the hindered rotations) lies outside our experimental frequency range, which stops at 450 cm^−1^. Thus, for the fits reported in Fig. 2*B*, the center frequency of the librational modes of the water confined in the cavity was fixed to 650 cm^−1^ as in bulk water (36). By closer inspection, the increase in absorption with increasing frequency from 180 to 400 cm^−1^ is smaller than in the case of bulk water: ΔΔ*α*(400 cm^−1^)/ΔΔ*α*(180 cm^−1^) = 1.02 to 1.10 for confined water, while ΔΔ*α*(400 cm^−1^)/ΔΔ*α*(180 cm^−1^) = 1.45 for bulk water. This is indicative of a blue shift of the librational mode, which can be attributed to a strong steric hindrance encountered by the librations of the water molecules in the proximity of the cage’s internal surface. A similar linewidth narrowing of the librational mode was found for water confined in nanoporous silica glasses, but in that case it exhibited a blueshift of the peak frequency itself due to interaction with the hydrophilic matrix (46).

The most interesting part of the THz spectra arises from the observation of an unperturbed center frequency of the intermolecular HB stretching mode of water confined in the Ga_4_L_6_^12−^ cage. Table 1 provides the values of ${\tilde{\nu }}_{0}$ and $w_{0}$ in which the stretch band is centered at 180 cm^−1^ for confined water, which is (perhaps surprisingly) not shifted with respect to the center frequency of bulk water at 293 K (181 cm^−1^). The intermolecular vibrations of the confined water are clearly red-shifted by ∼10 cm^−1^ with respect to the same mode for water cooled to its freezing point (38), and by ∼35 cm^−1^ with respect to water under high (∼10 kbar) hydrostatic pressures (Table 1 and *SI Appendix*, Fig. S3) (37). Thus, the nanoconfined water cannot be considered as cold or pressurized water either, but indicates a similar intermolecular HB strength like that of ambient water.

**Table 1.** Spectral parameters of the intermolecular stretching band of water confined in the Ga_4_L_6_ cage and bulk water at different thermodynamic conditions

Fit parameter, cm^−1^	Water inside Ga_4_L_6_	Water (293 K)	Water (273.2 K)	Water (10 kbar)
${\tilde{\nu }}_{0}$	180 (4)	181 (2)	193 (2)	216 (4)
$w_{0}$	249 (18)	537 (3)	557 (4)	542 (9)

Parameters are obtained by fitting a set of damped harmonic oscillators. The statistical 2σ error is given in parentheses. Details and the results of the fit can be found in *SI Appendix*, Tables S1 and S2, Fig. S3, and Text (see also refs. 37 and 38 for further details).

At the same time, the confined water shows a significant decrease in the damping of the intermolecular stretching mode, characterized as a significant narrowing of the linewidth with respect to bulk water at 293 K (Table 1). Any decrease in linewidth is an indicator for a decreased variance in the fast dynamics (36) (*SI Appendix*) and has also been ascribable to a reduced number of degrees of freedom, i.e., an entropic signature of a more restricted set of molecular configurations that are available (45). To place the linewidth of the nanoconfined water into perspective, we find that its value of *w*_0_ = 250 cm^−1^ is greatly reduced with respect to ambient, cold, and pressurized bulk water (∼540 cm^−1^), as well as with respect to the two hydration bands around the hydrophobic groups of alcohol chains and lightly supercooled water at 266.6 K that exhibit linewidths between 340 and 440 cm^−1^. Instead, the observed linewidth of the confined water interpolates between that observed for hexagonal ice (*w*_0_ = 80 to 220 cm^−1^) and clathrate hydrates and amorphous ice (*w*_0_ = 280 to 300 cm^−1^) (37, 38).

### Theoretical Results.

**Fig. 3.**
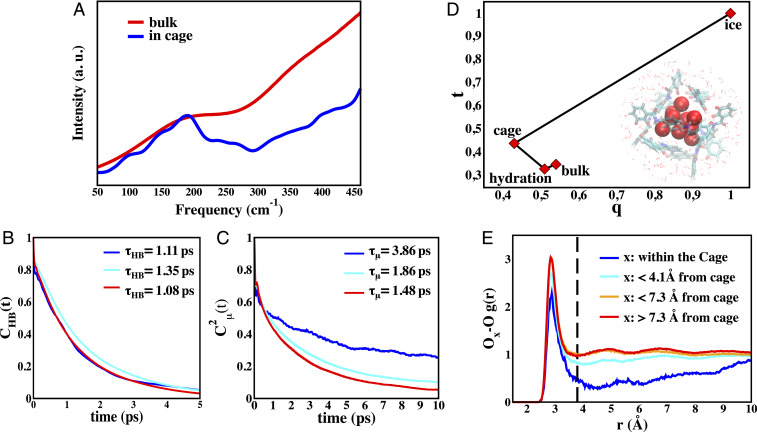
Water HB dynamics inside and outside the Ga_4_L_6_^12−^ cage. (*A*) Theoretical THz-IR spectra calculated for water inside the cage (blue) and for bulk liquid water taken from previous work (48) (red). The intensities are rescaled in order to have the same intensity for the maximum at ∼180 cm^−1^ to aid comparison. (*B*) Hydrogen-bonded lifetimes $C_{\mathrm{HB}}\left(t\right)$ and (*C*) orientation correlation $C_{\mu }^{\left(2\right)}\left(t\right)$ dynamics inside the cage (blue), in the hydration layer (cyan) and in the bulk liquid (red). The characteristic relaxation times are also reported in the legend (details on defined regions are given in *SI Appendix*, Text and Fig. S5). (*D*) The t and q order parameter space showing their values for cubic ice, water confined inside the cage, water in the hydration layer outside the cage (<4.1 Å), and bulk liquid water. The t parameter value for cubic ice (*t* = 1) is estimated for a fcc crystal (52), while that the *q* parameter (*q* = 1) is that of a perfect tetrahedral environment. The *Inset* illustrates the average position of the nine arrested water molecules with long residence time inside the cage. (*E*) Oxygen–oxygen radial distribution function *g*_OO_(*r*) for water inside the cage (blue) compared to the bulk (red).

To provide support for the experimental interpretations of the dynamics and structure described above, we have performed AIMD simulations of the solvated [Ga_4_L_6_]^12−^ host to characterize the encapsulated water molecules, using a well-characterized metageneralized gradient approximation (meta-GGA) functional B97M-rV (47), shown to describe bulk water well (48, 49). Fig. 3*A* provides the AIMD-simulated THz spectra of water inside the cage and the bulk water spectrum compared to experiment (*Materials and Methods*). The theoretical spectrum reproduces accurately the two main features of the THz measurements: 1) the same position of the intermolecular hydrogen-bonded stretching band at 180 cm^−1^ for both water inside the cage and in the bulk, and 2) the reduction in linewidth for water inside the cage with respect to bulk water. An AIMD additional simulation with the [Et_4_N]^+^ guest does not exhibit differences in interfacial properties near the cage or bulk (*SI Appendix*, Fig. S4), and thus does not contribute to the difference THz spectra.

Given the excellent agreement of the theoretical spectra with experiment, we can now analyze the trajectories to determine the time-averaged number of water oxygen centers inside the salt-free cage is 12.4 ± 0.7, whereas we find an average of 3.4 ± 0.6 water molecules inside the cage when filled with the cationic substrate. However, in the water-filled cage, there are 9 ± 1 water molecules that are dynamically distinct, with long residence times that exceed that of the 1- to 3-ps timescale of bulk water (50, 51) by at least an order of magnitude, and connect directly to what is observed experimentally. The remaining ∼3 waters undergo fast exchange dynamics with the bulk water at the interface (*SI Appendix*, Fig. S4). These “fast” waters are experimentally subtracted from the THz difference given in Eq. **1**, and thus lead to no contradiction with their physical presence in the cage from the simulation.

To quantify the motions for the water-filled Ga_4_L_6_^12−^ cage to connect to the THz observable, we evaluate the intermittent water–water HB autocorrelation function, $C_{\text{HB}}\left(t\right)$, as follows:

$$C_{\mathrm{HB}}\left(t\right)=\frac{\langle h\left(t\right)h\left(0\right)\rangle }{\langle h{\left(0\right)}^{2}\rangle },$$ [2]

where the operator h(t) is 1 when a given HB is intact and 0 otherwise (53). We find that the HB-lifetime $\tau_{\text{HB}}$ is very similar for all water regions ∼1 ps (Fig. 3*B*). This is in agreement with the experimental observation that the central frequency of the 180 cm^−1^ band is unshifted for water inside of the cage and the bulk. We have also calculated the orientational correlation function of the dipole vector of the water molecules as follows:

$$C_{\mu }^{\left(2\right)}\left(t\right)=\frac{\langle P_{2}\left[\mu \left(t\right)\cdot \mu \left(0\right)\right]\rangle }{\langle P_{2}\left[\mu \left(0\right)\cdot \mu \left(0\right)\right]\rangle },$$ [3]

where $P_{2}$ is the second-rank Legendre polynomial and μ(t) is the water dipole moment (unit vector) at time t (54, 55). Inspection of Fig. 3*C* reveals that water orientational dynamics is remarkably slower inside the cage when compared to the hydration and bulk water, in which the orientation relaxation time, $\tau_{\mu }^{\left(2\right)}$ is ∼2.5 times longer for water molecules in the host. The slowdown of water orientational dynamics inside the cage can be rationalized in terms of the constraints imposed by confinement on allowed reorientations and thus fewer hydrogen-bonded network configurations available within the cage with respect to the external solvent. This result is in agreement with the speculations made on the experimental side as regards the linewidth analysis of the spectral band at 180 cm^−1^, which is found to be much sharper for water inside the cage than for bulk liquid water. This signature is likely attributable to the lost translational motion as measured by long residence times as well as arising from restricted rotational motions for water in the cage.

To characterize the “phase” of water within the cage, as much as can be said for such a small cluster, we consider two popular structural order parameters used to describe the structure, dynamics, and thermodynamics of bulk water over its phase diagram (details are provided in *SI Appendix*) (56). Fig. 3*D* shows that water within the cage is different from the interfacial water near the nanocage interface, bulk (liquid) water, and ice. When considering the order parameters for water inside the cage, one can in particular notice that t is larger than for bulk liquid water, while the opposite is true for q. This t−q trend has been shown previously to occur when bulk water is isothermally compressed at a low temperature (56), and from this one would be tempted to correlate the phase of water inside the cage to that of pressurized water. This assumption can be checked by analyzing the $g_{\mathrm{OO}}\left(r\right)$ for water inside the cage and in the bulk as shown in Fig. 3*E*. In a recent work, it has been shown that when the pressure is increased on the water liquid, the four to five waters residing in the region of the first peak of $g_{\mathrm{OO}}\left(r\right)$ are nearly unchanged, whereas in the region beyond the first peak large structural changes occur with the collapse of the second hydration shell and shifting of higher shells to shorter distances (57). On the contrary, we find that $g_{\mathrm{OO}}\left(r\right)$ for water in the cage shows a less intense peak with respect to bulk water, with no significant density in the outer shells. From this, we can infer that water within the cage is not equivalent to pressurized water, despite the fact that they have some similarities in terms of t−q order parameters. When the aqueous Ga_4_L_6_^12−^ supramolecular tetrahedral assembly is simulated at a low temperature of 260 K, the conclusion that the water inside the cage is remarkably different from bulk water and ice does not change (*SI Appendix*, Fig. S5).

The t and q order parameters are summarized in *SI Appendix*, Table S3, for cubic ice, water confined inside the cage, water in the hydration layer outside the cage (<4.1 Å), and bulk liquid water. In *SI Appendix*, Table S3, we also report the water coordination number as defined by integration under the first peak of the *g*_OO_(*r*) at various cutoff values, as well as the number of HBs per molecule (53). All of the structural signatures (*SI Appendix*, Table S3) support that water molecules inside the cage are severely undercoordinated, with greatly reduced hydrogen bonding with respect to bulk liquid water. In particular, water inside the cage forms on average ∼1.8 HBs/molecule compared to ∼3.4 HBs/molecule formed in bulk liquid water. The water undercoordination suggests that, instead of an ice-like structure, water within the cage most likely behaves as an isolated small droplet with a fixed structure unlike other bulk phases. This is in agreement with the conclusion reached from the THz experiment, which finds that the encapsulated water does not resemble any testable phase of water, instead exhibiting mixed spectroscopic signatures of the liquid and solid phases over different parts of the water phase diagram.

## Discussion

Having supported the experimental conclusions, we next consider a simple thermodynamic model for the encapsulation process for solutes in the nanocage to estimate the solvation free energy changes. Our free energy approximation decomposes the solvation process into two steps, where the first step consists in creating a cavity in the liquid that can accommodate the ion, while the second step consists of putting a point charge at the center of the cavity (58). The free energy of the whole solvation process is then given by the sum of the free energy terms related to the two steps:

$$\Delta \mu =\Delta \mu_{\mathrm{cav}}+\Delta \mu_{\mathrm{ion}},$$ [4]

for which the first term $\left(\Delta \mu_{\mathrm{cav}}\right)$ is the energetic cost to form a cavity in the liquid that can accommodate the ion, and the second term in Eq. **4** refers to the energetic gain from the interactions between the point charge at the center of the cavity and the surrounding water molecules.

The cavitation free energy can be estimated from MD simulations by calculating the probability, $P_{v}\left(0\right)$, to observe 0 water molecules in a probe volume, v, as follows:

$$P_{v}\left(0\right)=e^{-\beta \Delta \mu_{\mathrm{cav}}},\;\text{where}\;\beta =1/k_{B}T.$$ [5]

**Fig. 4.**
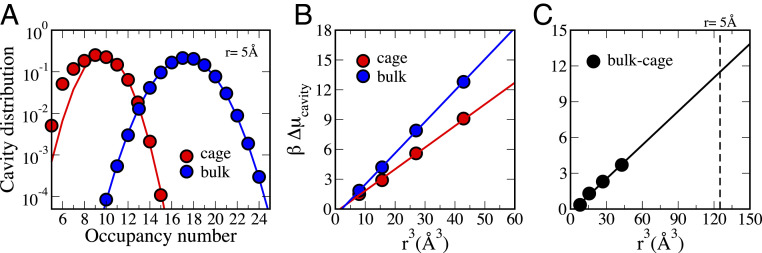
Cavity distributions and cavitation free energies for water in a sphere radius of 5.0 Å within the supramolecular cage and in the bulk. (*A*) The computed occupancy data for the cage (red) and bulk water (blue) are compared to Gaussian distributions (solid lines). The maximum probable occupancy number *n* = 9 observed for the 5.0-Å cavity within the cage is obtained in the bulk for a smaller cavity of 4.0-Å radius. (*B*) Cavitation free energy $\left(\Delta \mu_{\mathrm{cav}}\right)$ inside the cage (red) compared to bulk (blue) as a function of the cavity volume. The circles represent the calculated $\Delta \mu_{\mathrm{cav}}$ values for *r* = 2.0, 2.5, 3.0, and 3.5 Å, while the solid lines are linear fits. The linear trends indicate that $\Delta \mu_{\mathrm{cav}}$ scales with the cavity volume both inside the cage and in the bulk. (*C*) Difference between $\Delta \mu_{\mathrm{cav}}$ in the bulk and inside the cage as a function of cavity size, obtained by subtracting the red to the blue curve in *B*. The linear trend of the difference values vs. cavity volume allows to extrapolate to *r* = 5 Å (vertical dashed line, corresponding to the cavity formed by [Et_4_N]^+^). A value of 11.6 *k*_b_*T* is obtained from the extrapolation.

Using Eq. **5** and a spherical probe volume,v, with a radius of 5.0 Å, representative of the size of the [Et_4_N]^+^ guest, Fig. 4*A* shows that the maximum probable number of waters within the supramolecular cage corresponds to an occupancy *n* ∼ 9 water molecules, indicating that this cluster size is thermodynamically stable. The same occupancy number is obtained in bulk water for a much smaller observation volume (4.0 Å cavity in the bulk vs. 5.0 Å cavity inside the cage). Furthermore, the distributions are highly non-Gaussian with deviations for the nanocage that are consistent with a significant air–water interface inside the capsule like that observed for hydrophobic-water interfaces (58).

In Fig. 4*B*, we have extrapolated values of the cavitation free energy derived from the occupancy plots in Fig. 4*A*, in which it is evident that the formation of a cavity in the nanocage will be easier relative to bulk, which we estimate to be more favorable by ∼12 *k*_b_*T* (Fig. 4*C*). We note that the free energy cost to form small volume cavities (< 1 nm) in bulk water has been demonstrated to be mostly entropic (58–60), due to the constraints imposed on the hydration water network surrounding the cavity. In contrast, water molecules confined within the cage already have more constrained translational and rotational motions, and the entropic penalty of emptying the cavity within the cage is alleviated with respect to the bulk water by at least an order of magnitude at room temperature.

**Fig. 5.**
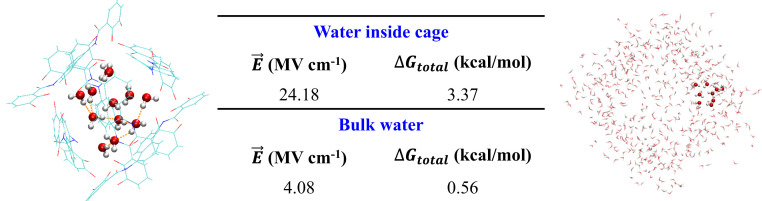
Electric field and free energies averaged over the water molecules inside the [Ga_4_L_6_]^12−^ assembly and in bulk water. We selected 20 snapshots characteristic of the equilibrated states from the AIMD trajectories in each case. The average free energy was evaluated as $<\Delta G^{†}>\;=\;\left(1/N\right)\sum_{i}0.048\cdot {\vec{\mu }}^{i}\cdot {\vec{E}}^{i}$ in which the unit conversion factor of 0.048 yields free energies in kilocalories per mole.

In addition to entropic effects, we also find that the greater electrostatic environment (10) destabilizes the HBs of the water cluster within the nanocage relative to bulk (Fig. 5), which would also contribute to an electrostatic preference for guest encapsulation. To this end, we have previously developed a field-bond-dipole model to quantify the average free energy for the HB:

$$<\Delta G>=\frac{1}{N}\sum_{i}{\vec{\mu }}^{i}\cdot {\vec{E}}^{i},$$ [6]

where the positive electric field sign convention is chosen for the projected electric field ${\vec{E}}^{i}$ on the *i*th HB that promotes electron flow for breaking the HB. We assumed an average dipole moment for the *i*th HB dipole moment ${\vec{\mu }}^{i}$ of 2.9 D consistent with previous ab initio studies of water that used Wannier centers to localize charge to water molecules to evaluate the dipole moment (61). We compared the average free energy estimated from Eq. **6** over the nine arrested water molecules inside the nanocage and compared against nine water clusters in the bulk water region as a control. While the electric field contribution for breaking HBs mostly averages out to destabilization energies comparable to *k*_B_*T* in the bulk liquid, as expected, the nanocage environment promotes more organized electric fields that significantly contributes to destabilization of hydrogen bonding in the water cluster in the nanocage.

The second term in Eq. **4** can be estimated from the simple Born solvation formula (62):

$$\Delta \mu_{q}=\frac{-q^{2}}{2R}\left(1-\frac{1}{\epsilon }\right),$$ [7]

where q is the charge of the ion, R is the cavity radius formed by the ion in water, and ϵ is the dielectric constant of water. We have previously noted the empirical nature of defining a cavity radius (63), and the value of the dielectric constant in the nanocage is of course uncertain at best and ill-defined at worse. However, if we make a reasonable assumption that the $\epsilon_{\mathrm{cage}}∼0.1\epsilon_{\mathrm{bulk}}$, the ion solvation free energy would be substantially unfavorable in the nanocage relative to the bulk. This explains why the nanocage itself needs to be highly charged to subsequently encapsulate the positively charged guest to overcome the large solvation free energy in the bulk liquid. Thus, encapsulation of positively charged guests such as [Et_4_N]^+^ is favored by the water structure within the cage in terms of free energy cost of cavity formation, and subsequently the large electrostatic stabilization afforded by the nanocage itself.

## Conclusion

In summary, this work is an experimental and theoretical characterization of the structure and dynamics of the water confined in the Ga_4_L_6_^12−^ tetrahedral assembly in solution under ambient conditions. Although we did not observe a shift of the intermolecular HB stretching center frequency with respect to bulk water at room temperature, indicating that the bond strength and length are not affected by confinement, the linewidth of this band is found to be about 55% smaller than in bulk water and more similar to that of amorphous ice or ice clathrates. This particular feature in the spectrum is a direct signature of the reduced number of degrees of freedom of the water molecules in confinement caused by a reduced number of available HBs to create a collective hydrogen-bonded network. Moreover, the librational motions of water, which are facilitated in a three-dimensional network since they involve a cooperative motion, as in the well-known jump mechanism (64), are restricted by the steric hindrance near the hydrophobic surface of the cavity.

The integrated results indicate that water confined in the Ga_4_L_6_^12−^ supramolecular host is not similar to water in any other thermodynamic state (e.g., at low temperature and/or high pressure), as also recently suggested by Heyden and Havenith (65) for proteins. Supporting AIMD simulations show that the dynamical signatures of the water droplet indicate that it is strongly arrested, and that it has a disrupted HB network. The simulations also support the spectroscopic interpretation of the narrowing of the linewidth of the intermolecular stretching mode due to reduced translational and rotational motions of the confined water.

This implies that any release of water from the host cavity into the bulk will be entropically favorable, supported by the more favorable cavitation of the water cluster in the supramolecular capsule relative to bulk. The release of the encapsulated waters is also enthalpically favored because the confined water cannot form as many HBs as in the bulk, and thus are “high energy” or “frustrated” (66–68), a result that is supported by the electric fields of the nanocage environment that destabilize HBs. The large charge of the nanocage itself is necessary to also drive encapsulation that contributes to the overall desolvation process of stripping off water molecules from the solvated reactant and subsequent preferential solvation of the transition state (69). In summary, the soluble Ga_4_L_6_^12−^ cage does create an inherently strong thermodynamic drive for guest encapsulation through desolvation of the host cavity (11–14, 66).

## Materials and Methods

The Ga-host synthesis has been reported previously (70, 71). The 1:1 binding with Et_4_N^+^ is verified by ^1^H-NMR of the synthesized host, which shows encapsulated Et_4_N^+^ and no free salt in solution (see *SI Appendix* for details about the sample preparation, measurements, and data analysis).

### THz Spectroscopy.

Spectra of Gallium supramolecular hosts aqueous solutions at 10 and 20 mM were recorded at 293 K in the frequency range from 50 to 450 cm^−1^ by THz-far infrared (THz) absorption spectroscopy. THz measurements were performed using a Bruker Vertex 80v Fourier-transform infrared spectrometer equipped with a liquid helium-cooled bolometer from Infrared Laboratories as detector. The sample solutions were placed in a temperature-controlled liquid transmission cell with polycrystalline diamond windows and a 25-μm-thick Kapton spacer. In total, 128 scans with a resolution of 2 cm^−1^ were averaged for each spectrum. The double difference absorption spectra were smoothened with a 2 cm^−1^ wide (5-point) moving average.

### Starting Geometries.

The starting geometry was built by removing the bis(trimethylphosphine) gold cation from Ga_4_L_6_^12−^ capsule of the reported X-ray structure (72), which was further fully optimized with density functional theory (DFT) in vaccum. The structure was then solvated using Gromacs with a preequilibrated normal density water box of size 30 × 30 × 30 Å. To maintain charge neutrality, K^+^ counter ions were also included for the encapsulated system. We ran an additional 3-ps AIMD simulation (300 K, 0.5-fs timestep) in the NVT ensemble to get further equilibration.

### AIMD.

All calculations presented in this paper were performed with DFT using the dispersion corrected meta-GGA functional B97M-rV (47, 73, 74) in combination with a DZVP basis set optimized for multigrid integration (75) as implemented in the CP2K software package (76, 77). In all cases, the simulated system consists of 2,572 atoms (including 760 water molecules) in a cubic box of 30 Å. We used periodic boundary conditions, five grids, and a cutoff of 400 Ry. Three independent AIMD simulations were performed for 30 ps in the NVE ensemble after an equilibration period of 6 ps (3 ps in the NVT ensemble with *T* = 300 K followed by 3 ps in the NVE ensemble). In the NVE trajectories, the average temperature was 318 ± 9 K. All results are based on averages over the three AIMD simulations. The time-averaged number of water inside the cage has been defined for each of the three independent simulations by counting at each step the number of waters within the cage and averaging over all MD steps.

### THz Spectra Simulation.

The theoretical IR spectra in the 50 to 500 cm^−1^ THz frequency range were calculated using the strategy developed recently based on the Fourier transform of the velocity–velocity correlation function modulated by atomic polar tensors (78, 79):

$$I\left(\omega \right)=\frac{2\pi \beta }{3cV}\sum_{u=x,\;y,\;z}\;\sum_{m=1}^{3N}\;\sum_{l=1}^{3N}\int_{-\infty }^{+\infty }dt\;e^{i\omega t}\left\langle P_{um}\left(t\right)v_{m}\left(t\right)P_{ul}\left(0\right)v_{l}\left(0\right)\right\rangle ,$$ [8]

where β=1/kT; ω is the frequency; *c*, the speed of light; *V*, the volume of the system; 〈⋯〉, the equilibrium time correlation function; *N*, the number of atoms of the system; and $v_{m}$, the *m*th element of the vvector that collects the 3*N* cartesian velocities of the *N* atoms of the system. $P_{um}=\partial \mu_{u}/\partial \xi_{m}$ is the *um* element of the atomic polar tensor, i.e., the first derivative of the *u*th component (*u* = *x*, *y*, *z*) of the total dipole moment M of the system with respect to the *m*th cartesian coordinate. The above equation takes into account all self- and cross-correlation terms, whether intramolecular or intermolecular, as well as both the charges and the charge fluxes contributions to the IR intensity, and simultaneously reduces the computational cost from the usual Fourier transform of the dipole moment correlation and accelerates signal convergence, without loss in accuracy (79). Velocities $\left(v_{m}\right)$ are readily obtained from the DFT-MD trajectories, while P(t) tensors have been parameterized on reference water structures (78). The spectra are calculated including water contributions only, and neglecting contribution from the cage and counter ions. By selecting the Cartesian coordinates of the atoms belonging to a specific vibrational population (class of water molecules with common structural and spectroscopic properties) into the summation in Eq. **4**, one gets the individual contribution of the selected population to the IR spectrum by reducing the summation over *m* = 1,3*N* to *m* = 1,3N*, where N* = 9 identifies the waters inside the cage.

## Supplementary Material

Supplementary File

## Data Availability

All study data are included in the article and *SI Appendix*.
