# Supplementary material for: An isolated water droplet in the aqueous solution of a supramolecular tetrahedral cage
Source: Proc Natl Acad Sci U S A. 2020 Dec 14;117(52):32954–61. doi: 10.1073/pnas.2012545117 (PMC7777103; doi:10.1073/pnas.2012545117)
Supplement: Supplementary File [file pnas.2012545117.sapp.pdf]

SUPPORTING INFORMATION FOR

An Isolated Water Droplet in the Aqueous Solution of a Supramolecular Tetrahedral Cage

Federico Sebastiani,[1] Trandon A. Bender,[2] Simone Pezzotti[1], Wan-Lu Li[2,3], Gerhard Schwaab,[1] Robert G. Bergman,[2] Kenneth N. Raymond,[2] F. Dean Toste,[2] Teresa Head-Gordon[2,3], Martina Havenith[1]

[1] Lehrstuhl für Physikalische Chemie II, Ruhr-Universität Bochum, 44780 Bochum, Germany
[2] Chemical Sciences Division, Lawrence Berkeley National Laboratory, and Department of Chemistry, University of California, Berkeley, California 94720-1460
[3] Pitzer Center for Theoretical Chemistry, University of California, Berkeley, California 94720-1460

*Corresponding authors: Robert G. Bergman, Kenneth N. Raymond, F. Dean Toste, Teresa Head-Gordon, Martina Havenith

Email:
*rbergman@berkeley.edu
*raymond@socrates.berkeley.edu
*fdtoste@berkeley.edu
*martina.havenith@rub.de
*thg@berkeley.edu

THz-FIR spectroscopy

THz-Far Infrared (THz-FIR) absorption measurements have been performed by means of a Bruker Vertex 80v Fourier Transform InfraRed (FTIR) spectrometer equipped with a liquid helium cooled silicon bolometer from Infrared Laboratories as detector. A mercury arc lamp was used as light source of the broadband THz radiation, while a Mylar-multilayer beamsplitter was employed for the generation of the interferogram. A sketch of the experimental setup is shown in Figure S1.

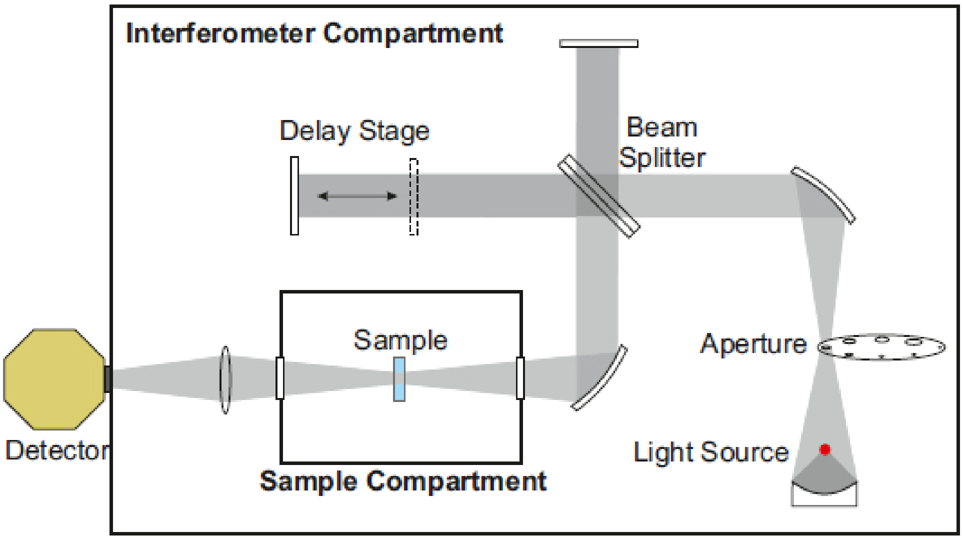

Figure S1: *Setup schematic of the FTIR spectrometer*. THz radiation from a mercury arc lamp is directed through an aperture of adjustable size to a Michelson interferometer, consisting of a beam splitter, a fixed mirror and a mirror on a delay stage. The two interfering beams are directed through the sample onto the detector, which records the interferogram.

The sample compartment was continuously purged with N_2 gas to minimize atmospheric water vapor lines in the signal beam path and the degradation of the samples. The data reported here were recorded using a temperature controlled sample cell at a temperature of 293 ±0.2 K and at a humidity level below 5%. For these measurements we used a Bruker liquid cell with chemical vapor deposition-grown diamond windows (Diamond Materials, GmbH). The sample layer thickness is fixed by Kapton spacers in between the windows (the nominal thickness was 25 μm). During the measurements, the precise layer thickness was determined from the interference pattern of the empty cell by mid-infrared absorption spectroscopy. Each sample was measured in the frequency range from 50 to 450 cm^{-1} (1.5-12 THz) as an average over 128 scans with a typical resolution of 2 cm^{-1}.

The samples were prepared as described in the other Section and dissolved in degassed ultrapure water in an oxygen-free environment right before each measurement. The mass density of the solutions at 293 K was measured by an Anton-Paar DMA58 density meter to calculate accurately the concentration of solutes and solvent in all the samples.

Data Analysis of Concentration-dependent Absorption Spectra

Using Lambert-Beer's Law, the frequency and temperature-dependent absorption coefficient of an aqueous solution ($\alpha_{solution}(\nu)$) is expressed as:

$$\alpha_{solution}(\nu) = \frac{1}{d} \ln\left(\frac{I_{water}(\nu)}{I_{solution}(\nu)}\right) + \alpha_{water}(\nu)\,, \qquad \text{(S1)}$$

where d is the sample thickness, $I_{water}(\nu)$ and $I_{solution}(\nu)$ are the experimental transmitted intensities of the water reference and the sample at temperature T, respectively. $\alpha_{water}(\nu)$ is absorption coefficient of bulk water and is obtained from a fit of the spectrum of water at a given temperature T (see next Section). Using water as a reference, artifacts due to the reflections at the cell windows are minimized. The remaining absorption due to residual air in the absorption path was corrected by taking into account a scaled spectrum of water vapor.

The effective absorption of the solute and its hydration water with respect to the bulk liquid can be deduced from the following equation:

$$\Delta\alpha(\nu) = \alpha_{solution}(\nu) - \frac{c_w}{c_w^0}\alpha_{water}(\nu), \quad \text{(S2)}$$

where c_w and c_w^0 are the water concentrations in the solution and in bulk water as obtained from mass density measurements at room temperature (T=293 K), respectively. This procedure allows us to remove nonlinearities caused by the solute concentration-dependent change of the apparent molar volume, as described in Ref.[1].

In the case of Gallium supramolecular clusters (Ga-clusters), we also did a double difference ($\Delta\Delta\alpha(\nu)$) between the effective absorption of the clusters with and without guest molecules, referencing both of them to bulk water. This yields:

$$\Delta\Delta\alpha(\nu) = \Delta\alpha(\text{Ga} - \text{clusters})(\nu) - \Delta\alpha(\text{Ga-clusters+Et}_4\text{NCl})(\nu) \quad \text{(S3)}$$

where $\Delta\alpha(\text{Ga} - \text{clusters})(\nu)$ and $\Delta\alpha(\text{Ga} - \text{clusters})(\nu)$ are the effective absorption coefficients of the Ga-clusters at room temperature (T=293 K) without and with Et_4NCl with respect to bulk water, respectively, as defined in Equation (S2).

Model of Bulk Water Absorption Coefficient

The absolute absorption coefficient ($\alpha_{water}(\nu)$) of water at T=293 K was obtained by referencing a set of temperature-dependent THz absorption measurements taken in our laboratory to the water data obtained by Bertie and Lan [2]. The corresponding bulk extinction coefficient at T=293 K ($\varepsilon_{water}(\nu)$) was fitted to one Debye term and three damped harmonic oscillator terms:

$$\varepsilon_{water}(\nu) = \frac{\alpha_{water}(\nu)}{c_w^0} = \varepsilon_{LF}(\nu) + \varepsilon_{MID}(\nu) + \varepsilon_{HF}(\nu) \quad \text{(S4)}$$

where

$$\varepsilon_{LF}(\nu) = \frac{\tilde{a}_0 \exp\left(-\frac{\nu}{\tilde{\nu}_{co,i}}\right)}{\pi\left(\nu^2 + \frac{\tilde{w}_0(0)^2}{\pi^2}\right)} \quad \text{(S5a)}$$

$$\varepsilon_{MID}(\nu) + \varepsilon_{HF}(\nu) = \sum_{i=1}^{3} \frac{\tilde{a}_i w_i^2(\nu)}{4\pi^3\left(\left(\tilde{\nu}_{b,i}^2 + \frac{w_i^2(\nu)}{4\pi^2} - \nu^2\right) + \frac{\nu^2 w_i^2(\nu)}{\pi^2}\right)}\ \nu^2 \quad \text{(S5b)}$$

And $\tilde{w}_i(\tilde{\nu}) = \tilde{w}_i(0)\exp\left(-\frac{\nu}{\tilde{\nu}_{co,i}}\right)$ is a frequency dependent damping factor. The bulk water parameters are given in Ref.[1]. This model was used in the main text to estimate the number of water molecules in the supramolecular hosts. The total error of the model in Eqs.(S4-S5b) is lower than 5%. In Figure S2, the comparison between this model for different numbers of water molecules and the experimental data ($\Delta\Delta\alpha(\nu)$) at T=293 K is reported.

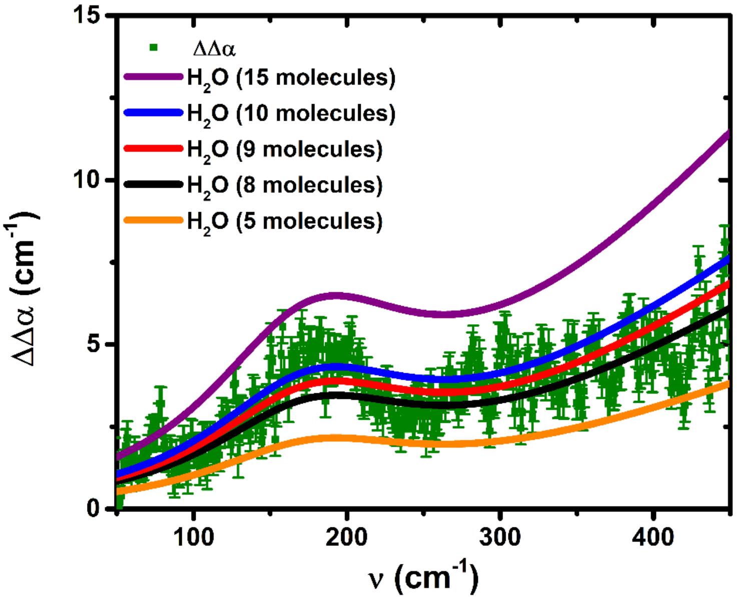

Figure S2: *Absorption spectrum of water confined in Ga-clusters.* $\Delta\Delta\alpha(\nu)$ at 10 mM (green points), and water absorption spectra of bulk water at 293 K for different numbers of water molecules (purple, blue, red, black and orange lines).

Fit of the Concentration-dependent Absorption Spectra

The following fitting functions were employed for the dissection of the $\Delta\Delta\alpha(\nu)$ and were adapted from the general model for effective absorption of aqueous solutions in the THz-Far Infrared range [1]:

$$\Delta\Delta\alpha(\nu) = n_{LF}\varepsilon_{LF} + \sum_{n=1}^{N} \frac{\alpha_n w_{0,n}^2 \nu^2}{4\pi^3\left[\frac{\nu^2 w_{0,n}^2}{\pi^2} + \left(\tilde{\nu}_{d,n}^2 + \frac{w_{0,n}^2}{4\pi^2} - \nu^2\right)^2\right]} + n_{HF}\varepsilon_{HF} \qquad \text{(S6)}$$

The first term $n_{LF}\varepsilon_{LF}$ was introduced to describe the Debye relaxations of water at the given temperature at low frequency (below 50 cm^{-1}), while the last term accounts for the librational modes of water (above 300 cm^{-1}). n_{LF} and n_{HF} are the corresponding scaling factors used as fit parameters. In this specific case, the second term is due to N water modes, that are described by damped harmonic oscillators with a_n, $w_{0,n}$, and $\tilde{\nu}_{d,n}$ describing the amplitude, the width and the center frequency of the nth-resonance. The corresponding center frequency of an

unperturbed Brownian harmonic oscillator can then be deduced accordingly: $\tilde{\nu}_{0,n} = \sqrt{\tilde{\nu}_{d,n}^2 + \frac{w_{0,n}^2}{4\pi^2}}$ and it is shifted compared to $\tilde{\nu}_{d,n}$ by a relaxation with the damping width ($w_{0,n}$). The unperturbed center frequency is also directly related to the strength of the bond involved in the vibrational mode as $\tilde{\nu}_{0,n} = \frac{1}{2\pi}\sqrt{\frac{k}{\mu}}$, where k is the effective force constant and μ is the reduced mass, respectively. Hereafter, the subscript n in the fitting parameters is omitted for the sake of clarity.

Table S1: *Fitting parameters of ΔΔα for Ga-clusters at 10 mM and of the molar extinction of bulk water at T=293 K at ambient pressure (this work) and at 10 kbar [3], and T=273.2 K [5].* The perturbed and unperturbed center frequencies ($\tilde{\nu}_d$ and $\tilde{\nu}_0$, respectively) and the linewidths (w_0) are given in cm^{-1}, while the lifetime is given in fs.

sample	$\tilde{\nu}_d$ (cm^{-1})	$\tilde{\nu}_0$ (cm^{-1})	w_0 (cm^{-1})	τ (fs)
**Ga-clusters at 293 K**	176 ± 2	180 ± 4	249 ± 18	134 ± 10
**Bulk water at 293 K**	159 ± 1	181 ± 2	537 ± 3	62 ± 1
**Bulk water at 273.2 K**	171 ± 1	193 ± 2	557 ± 4	60 ± 4
**Bulk water at 10 kbar**	198 ± 1	216 ± 4	542 ± 9	62 ± 1

Moreover, in the homogenous approximation, the lifetime of the oscillator's dipole moment autocorrelation function (τ), i.e. the lifetime of the excited resonances, is inversely related to the damped harmonic oscillator linewidth (w_0) and the speed of light (c) [1], as: $\tau = \frac{1}{w_0 c}$.

ΔΔα for Ga-clusters at 10mM and of the molar extinction for bulk water at 273.2 K and 293 K were fitted, using the function defined in Equation (S7). The results of the fit for the intermolecular stretching band are reported in Table S1, together with those for water under high hydrostatic pressure [3]. The uncertainty on the fitted parameters correspond to 2σ standard errors, i.e. we have a confidence level of 95%.

A comparison of the fitted inter-molecular stretching band for Ga-clusters at 10mM and 20mM and bulk water at 293 K is reported in Figure 2 in the main text. The same model was applied to the absorption spectrum of hexagonal ice, low-density amorphous ice and supercoooled water at 266.6 K [4, 5]. The results are listed in Table S2 and shown in Figure S3. On the basis of the measured linewidths, we deduce lifetimes of 130 fs for an excited dipole belonging to the intermolecular stretching of the hydrogen bonds of the water encapsulated in the supramolecular host, as reported in Table S1. The latter value is larger than that found in

bulk water at different thermodynamic conditions (including supercooled water), but smaller than that of ice (see Table S1 and S2). τ is known to be correlated to the distribution of thermal bath states and their coupling to the oscillator under consideration. Upon confinement, the effective reduction of the lifetime with respect to bulk water indicates a narrower distribution of accessible states. [1]

Table S2: *Fitting parameters of the absorption coefficient (α) for hexagonal [5] and amorphous ice (this work) and supercooled water at 266.6 K [5].* The perturbed and unperturbed center frequencies ($\tilde{\nu}_d$ and $\tilde{\nu}_0$, respectively) and the linewidths (w_0) are given in cm^{-1}, while the lifetime is given in fs. The statistical 2σ error are also given.

$\tilde{\nu}_d$ (cm^{-1})	$\tilde{\nu}_0$ (cm^{-1})	w_0 (cm^{-1})	τ (fs)
**Hexagonal Ice (Ih)**			
79 ± 3	72 ± 3	207 ± 30	160 ± 23
152 ± 10	150 ± 9	132 ± 11	252 ± 21
192 ± 20	191 ± 20	125 ± 32	265 ± 66
216 ± 5	215 ± 5	80 ± 8	416 ± 40
241 ± 32	238 ± 32	221 ± 15	151 ± 10
337 ± 36	335 ± 36	207 ± 30	160 ± 23
**Amorphous Ice**			
86 ± 3	84 ± 2	110 ± 19	303 ± 52
155 ± 2	148 ± 1	281 ± 20	119 ± 8
218 ± 1	212 ± 1	306 ± 12	109 ± 4
294 ± 3	291 ± 2	263 ± 31	127 ± 15
**Supercooled Water (at 266.6 K)**			
148 ± 3	129 ± 3	456 ± 10	73 ± 2
203 ± 2	189 ± 2	456 ± 10	73 ± 2
529 ± 3	489 ± 3	1256 ± 3	27 ± 1

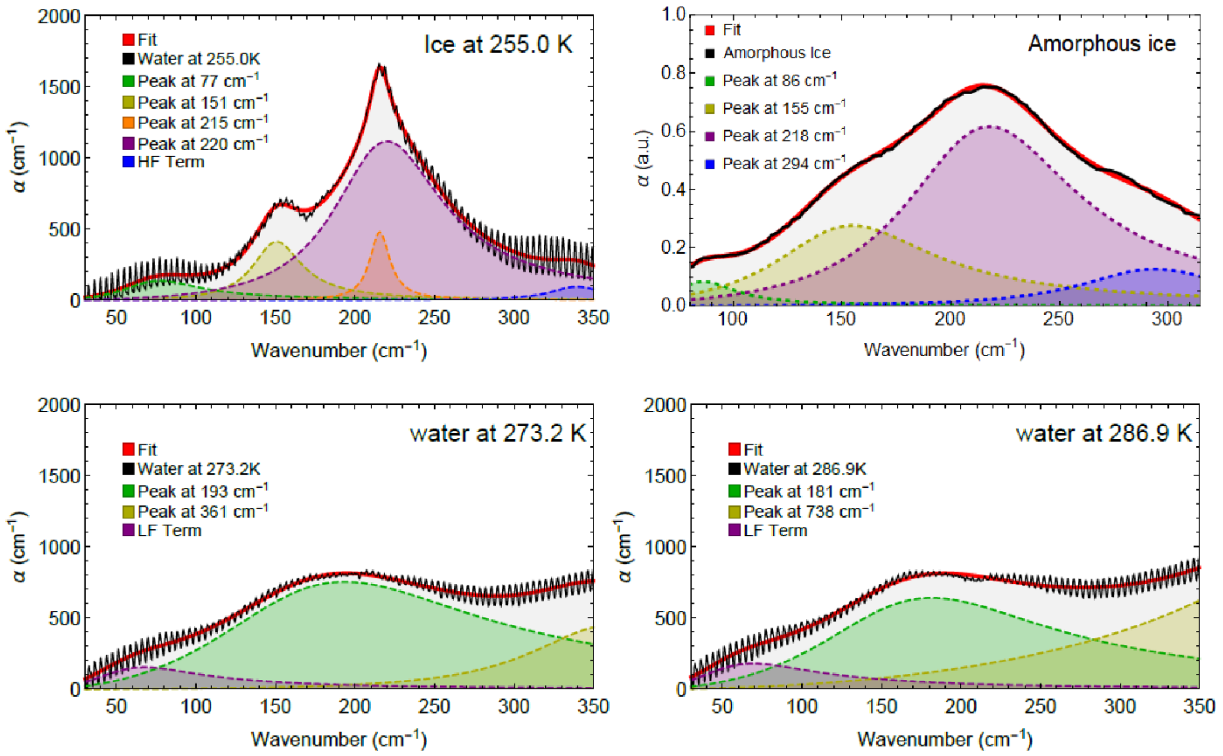

Figure S3. *Absorption spectra of Ice Ih, low-density amorphous ice, water at 273.2 K and of supercooled water at 266.6 K at ambient pressure.* Each spectrum (black line) was modeled by a sum of damped harmonic oscillator functions (red line), as described in the text. The individual harmonic oscillators are color-coded using the same colors for similar center frequencies for the top and bottom panels. Reproduced and adapted from Ref.[5], with the permission of AIP Publishing. Data in the top-right panel are from Ref.[4] and fitted with the model described in the text.

AIMD Results for $[Ga_4L_6]^{12-}$ Host Filled with Water vs. $[Et_4N]^+$ Substrate

In comparisons to the pure bulk liquid for the THz simulation, we utilize the data provided in Refs.[6, 7]. In this way, the theoretical THz signatures from water inside the cage are directly compared to that of pure bulk liquid water, as done experimentally (see Figure 2). For the structural and dynamical analysis of the bulk region in the solution with counterions outside the $[Ga_4L_6]^{12-}$ tetrahedral assembly, we determined the reference for bulk solution as follows. In Figure S4A, the vertical dashed lines identify water layers: water within 4.1 Å from the cage (blue dashed line) forms the hydration layer around the cage, while water farther than 4.1 Å from the cage shows bulk-like coordination and t-q order parameters. Averages over all water molecules in the designated bulk region provide a reference radial distribution functions (Figure 3E), as well as water coordination number and number of hydrogen-bonds per molecule (Table 2), that can be compared to water in and at the interface of the cage. The hydrogen-

bonds are defined using the standard distance and angle criterion from Luzar [8], with O-O distance cut-off of 3.5 Å and H-O--O angle in the 0-30° range.

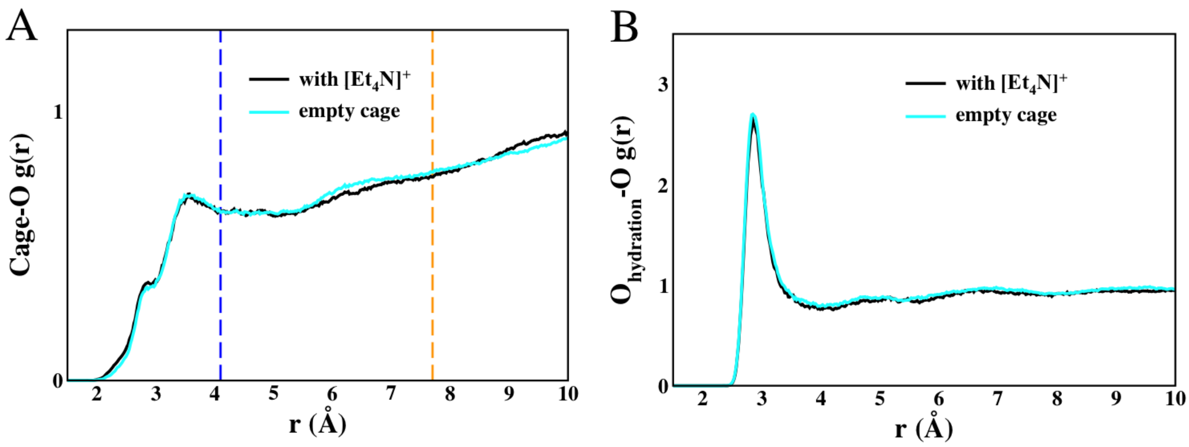

Figure S4: *Radial distribution function g(r) in between all atoms of the cage and all O atoms of water molecules, with and without the $[Et_4N]^+$ substrate.* (a) Water within 4.1 Å from the cage (cyan), within 7.3 Å from the cage (orange). (b) rdf between the O-atoms of water molecules in the hydration layer (within 4.1 Å from the cage, as marked by the blue dashed line in panel A and discussed in the main text) and the O-atoms of all water molecules.

In order to check how the binding with the substrate affects the properties of water outside the cage, and thus contributes to a component to the difference THz spectra, an additional AIMD simulation was performed with the same computational setup (see Methods) with the $[Et_4N]^+$ substrate inside the cage. The system was equilibrated at T=300 K for 5 ps, and a subsequent trajectory was accumulated for 10 ps for further analysis. The shorter simulation time as compared to the 30 ps simulations is still sufficient to sample the structural properties of interest in the present work. In Figure S4 we compare the rdf calculated between water molecules in the hydration layer and all other water molecules, for the simulations with the $[Et_4N]^+$ substrate within the cage or filled with water, in which no significant differences are observed. Hence the binding of the substrate within the cage has little effects on hydration water properties outside the cage is obtained from the evaluation of t and q order parameters, which do not differ with that reported in Table S3.

As regards the number of water molecules inside the cage, we find that the average of 12.4±0.7 waters instantaneously located within the cage is reduced to 3.4±0.6 in presence of the guest. This means that the difference of the average number of waters inside the cage with and without the guest is 12.4±0.7 - 3.4±0.6 = 9.0±0.7, in excellent agreement with the experimental estimation of 9-10 water molecules. Moreover, when the guest is not present, as

discussed in the main text, only 9-10 (9 in two simulations and 10 in one simulation) of the 12.4±0.7 waters have long residence times inside the cage (>96% of simulation time spent within the cage), while the other 3 molecules, as well as the 3.4±0.6 waters remaining also in presence of the guest, continuously enter and exit the cage (more than 6 inside/outside exchanges for each of these waters in 10 ps).

Table S3: *Hydrogen bonding and order parameters.* Hydrogen bond index per molecule, coordination number (CN) with different cutoff values of the first shell, orientational (q) and translational (t) order parameters for bulk water, interfacial water at the cage surface and water inside the cage.

Water System	HBs per molecule	CN (3.2 Å)	CN (3.8 Å)	q	t
bulk	3.4	3.8	6.6	0.54	0.35
hydration	2.9	3.4	5.8	0.50	0.33
cage	1.8	2.8	4.5	0.43	0.44

AIMD results for order parameters for water in the $[Ga_4L_6]^{12-}$ host and bulk water

To analyse the "phase" of the water droplet we evaluate the translational order parameter, t, defined as

$$t = \frac{\int_0^{\xi_c} d\xi \; |g_{OO}(\xi) - 1|^2}{\xi_c} \tag{S7}$$

where $g_{OO}(\xi)$ is the oxygen-oxygen radial distribution function, $\xi = r\rho^{1/3}$, r is the distance between the oxygen atoms of a pair of molecules, ρ is the bulk water density, and ξ_c is a cut-off distance that we set to 3 Å in this work. For an ideal gas, g(ξ) = 1 everywhere and t vanishes, whereas in a crystal there is long-range translational order, and g(ξ) ≠ 1 over long distances and hence t is large; for example, values of t =1 can be obtained for an fcc crystal such as cubic ice. [9] We also evaluate the q parameter

$$q = 1 - \frac{3}{8} \sum_{i=1}^{3} \sum_{j=i+1}^{4} \left(cos\phi_{ij} - \frac{1}{3} \right)^2 \tag{S8}$$

measures tetrahedral order, where $cos\phi_{ij}$ is the angle formed by the lines joining the oxygen atom of a given molecule and those of its nearest i,j neighbours (≤ 4). The average value of q varies between 0 (in an ideal gas) and 1 (in a perfect tetrahedral network, as it is the case for ice).

Temperature Effect: 320 K vs 260 K

To check the effect of temperature on water inside and outside the $[Ga_4L_6]^{12-}$ tetrahedral assembly, an MD simulation was performed in the NVT ensemble with T=260 K. The initial configuration was taken from the 320 K simulations and further equilibrated at the new temperature for 5 ps, until it stabilized around 260 K. After equilibration, the trajectory was accumulated for 13 ps for further analysis. We find that the lower temperature yields no detectable influence on the water population in the cage compared to ambient (Table S4), and the average number of water molecules hydrating the cage is unchanged. Accordingly, we also find that the average number of water molecules within the cage, as well as the number of waters with long residence time, has negligible temperature dependence.

Table S4: *Characterization of water analysis for the water filled $[Ga_4L_6]^{12-}$ tetrahedral assembly at 260 K vs 320 K.* N^*_{cage} refers to the number of water molecules with long residence time inside the cage, N_{cage} is average number of water molecules physically in the cage, N_{hyd} is the average number of water molecules in the hydration layer. The q_i and t_i order-parameters for water in $i=$ cage, hydration, and bulk regions (see main text for definitions).

Temperature	N^*_{cage}	N_{cage}	N_{hyd}	q_{cage}	t_{cage}	q_{hyd}	t_{hyd}	q_{bulk}	t_{bulk}
320 K	9-10	12.4 ± 0.7	189.9	0.43	0.44	0.50	0.33	0.54	0.35
260 K	10	11.7± 0.6	191.1	0.45	0.52	0.56	0.39	0.60	0.40

Analysis of the q and t order parameters for water in the cage/hydration/bulk regions reveals that water behaviour is systematically shifted slightly in increased translational and orientational order at the lower temperature. Figure S5 shows the lower temperature does not revise the main conclusion that the water inside the cage is still remarkably different from bulk water and ice.

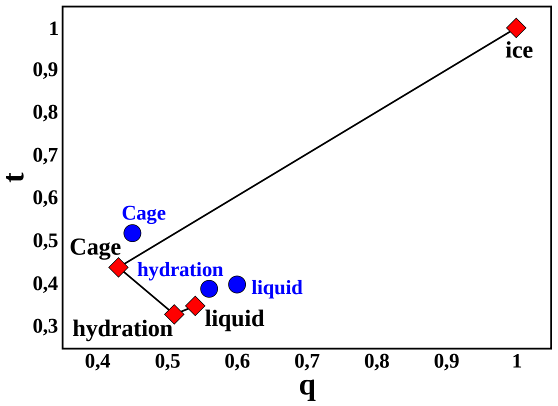

Figure S5. *The q and t order-parameters.* The order-parameter space (q-t) of the fcc ice, water inside the cage, water in the hydration layer outside the cage (<4.1 Å), and the bulk liquid water at temperatures 260 K (blue) and 320 K (red).

References:

[1] G. Schwaab, F. Sebastiani, M. Havenith, *Angew. Chem. Int. Ed.* **2019**, 58, 3000-3013.

[2] J. E. Bertie and Z. Lan, *Appl. Spectrosc.* **1996**, 50, 1047–1057.

[3] H. Vondracke, S. Imoto, G. Schwaab, D. Marx, M. Havenith – *J. Phys. Chem. B* **2019**, 123, 7748-7753.

[4] J-M. Zanotti, P. Judeinstein, S. Dalla-Bernardina, G. Creff, J-B. Brubach, P. Roy, M. Bonetti, J. Ollivier, D. Sakellariou M-C. Bellissent-Funel, *Sci. Rep.* **2016**, 25938.

[5] S. Funke, F. Sebastiani, G. Schwaab, M. Havenith, *J. Chem. Phys.* **2019,** 150, 224505.

[6] L.R. Pestana, N. Mardirossian, M. Head-Gordon, T. Head-Gordon, *Chem. Sci.* **2017**, *8*, 3554-3565.

[7] L.R. Pestana, O. Marsalek, T.E. Markland, T. Head-Gordon, *J. Phys. Chem. Lett.* **2018**, *9*, 5009-5016.

[8] A. Luzar, *J. Chem. Phys.* **2000**, 113, 10663–10675.

[9] T.M. Truskett, S. Torquato, P.G. Debenedetti, *Phys. Rev. E* **2000**, 62, 993-1001.
